# Increased PD-L1 and T-cell infiltration in the presence of HLA class I expression in metastatic high-grade osteosarcoma: a rationale for T-cell-based immunotherapy

**DOI:** 10.1007/s00262-016-1925-3

**Published:** 2016-11-16

**Authors:** Yayan T. Sundara, Marie Kostine, Arjen H. G. Cleven, Judith V. M. G. Bovée, Marco W. Schilham, Anne-Marie Cleton-Jansen

**Affiliations:** 1 Department of Pathology, Leiden University Medical Center, P.O. Box 9600, 2300 RC Leiden, The Netherlands; 2 Department of Rheumatology, Hôpital Pellegrin, Centre Hospitalier Universitaire de Bordeaux, Bordeaux, France; 3 Department of Pediatrics, Leiden University Medical Center, P.O. Box 9600, 2300 RC Leiden, The Netherlands

**Keywords:** Osteosarcoma, HLA class I, Tumour-infiltrating lymphocytes, PD-L1, Immunotherapy

## Abstract

**Introduction:**

Immunotherapy may be an excellent choice for treating osteosarcoma given its exceptionally high genomic instability, potentially generating neoantigens. In this study, we aim to investigate the HLA class I expression, PD-L1 and tumour-infiltrating lymphocytes in primary osteosarcomas and relapses/metastases, as well as their changes during disease progression.

**Materials and methods:**

Tumour samples from multiple stages of the disease (pretreatment biopsies, surgical resections of primary osteosarcomas, relapses and metastases) were collected and stained for HLA-A (HCA2), HLA-B/C (HC10), β2-microglobulin and PD-L1 using immunohistochemistry on whole sections. Density and type of T-cell infiltrate were characterised by a triple immunofluorescent staining CD3-CD8-FOXP3.

**Results:**

Overall, 85 formalin-fixed, paraffin-embedded blocks from 25 osteosarcoma patients were included. HLA class I expression was detected in 94% of osteosarcomas (strongly positive in 56%, heterogeneous in 38%) and negative or weakly positive in 6%, without differences between the stages of the disease. HLA-A expression was more frequently negative than HLA-B/C. Tumour-infiltrating lymphocytes were highly heterogeneous and mainly observed in tumour areas with expression of HLA class I. Density of T cells was significantly higher in metastases than in primary tumours and local relapses (*p* = 0.0003). Positive PD-L1 expression was found in 13% of primary tumours, 25% of relapses and 48% of metastases and correlated with a high T-cell infiltrate (*p* = 0.002).

**Conclusion:**

An increased number of tumour-infiltrating T cells and PD-L1 expression in metastases compared with primary tumours, suggesting accessibility for T cells, could imply that osteosarcoma patients with metastatic disease may benefit from T-cell-based immunotherapy.

**Electronic supplementary material:**

The online version of this article (doi:10.1007/s00262-016-1925-3) contains supplementary material, which is available to authorized users.

## Introduction

Osteosarcoma is the most common primary high-grade bone malignancy, primarily affecting children and adolescents [1]. It commonly arises in the metaphyseal plates of the long bones of the extremities (i.e. distal femur, proximal tibia), while tumours developing in the axial skeleton, pelvis or craniofacial bones tend to occur in older individuals. Osteosarcoma patients are treated with curative intent, consisting of surgery of the primary tumour and any resectable metastatic lesions, in addition to pre- and post-operative chemotherapy [2]. This multimodal therapeutic approach greatly improves the disease-free survival probability, from 10 to 20% with the surgery alone, to more than 60%. However, survival for patients with relapsed or metastatic disease remains dismal and unchanged over the last three decades, as efforts in developing novel active agents have been generally disappointing [3, 4]. Osteosarcoma’s genomic complexity is one of the major explanations for the lack of specific targetable mutations or molecular pathways. On the other hand, this genomic characteristic and the associated high mutational burden may generate specific tumour neoantigens, therefore providing potential targets for T-cell-based immunotherapies [5–7].

Historically, the first successful example of immunotherapy was in 1891 when William B. Coley injected a mixture of streptococcal bacteria into unresectable bone sarcomas, resulting in an immunological reaction and tumour regression [8, 9]. Recent advances in cancer immunology have now revealed the importance of a spontaneous antitumour immune reaction to predict response to immunotherapy, mostly carried out by cytotoxic CD8-positive T cells [10]. Antigen presentation by surface HLA expression on tumour cells is an important prerequisite for antitumour immunity since loss or down-regulation of HLA molecules is a common mechanism deployed by tumour cells to escape immune surveillance [11]. Another immune escape mechanism is the dysregulation of immune checkpoint pathways such as PD-1/PD-L1 axis, which has been actively studied in epithelial malignancies [12]. So far, data regarding the immune microenvironment in osteosarcoma are rather limited. In order to evaluate the feasibility of T-cell-mediated immunotherapies, we assessed HLA class I expression, PD-L1 and T-cell infiltration, as well as their changes during osteosarcoma progression using immunohistochemistry.

## Materials and methods

### Patient material

Formalin-fixed, paraffin-embedded materials from osteosarcoma patients with a known stable (*n* = 7) or progressive disease (*n* = 18) diagnosed between 1998 and 2011 were retrieved from the archives of the Pathology Department of the Leiden University Medical Center (LUMC). Patients characteristics are described in Table 1. Overall, 85 tissue blocks from 25 patients were collected for this study, including pretreatment biopsies (*n* = 16) and surgical resections of primary tumours (*n* = 18), local relapses (*n* = 25) and metastases (*n* = 26). Material from multiple stages of the disease was available for 17 patients, and five patients had metastatic disease at diagnosis (Supplementary Table 1). Whole sections were used to assess HLA class I expression, PD-L1 and T-cell infiltrate in these tumours. Diagnoses were confirmed by an experienced bone and soft-tissue tumour pathologist (J.V.M.G. Bovée) according to the 2013 World Health Organization classification. All the specimens were handled in a coded fashion according to the Code for Proper Secondary Use of Human Tissue in the Netherlands of the Dutch Federation of Medical Scientific Societies.

**Table 1:** Patient characteristics

*n*	25
Median age (years)	18 (7–70)
Gender	
Female	8 (32%)
Male	17 (68%)
Pre-operative chemotherapy	
Yes	20 (80%)
No	5 (20%)
Histological response	
Good	14 (56%)
Poor	6 (24%)
Unknown	5 (20%)
Median follow-up (months)	56 (14–117)
Progressive disease	
No	7 (28%)
Yes	18 (72%)
Metastases at time of initial diagnosis	
No	20 (80%)
Yes	5 (20%)

### Immunohistochemistry and scoring

Immunohistochemistry stainings for HLA class I (HCA2, HC10 and β2-microglobulin) and PD-L1 were performed according to standard laboratory protocols, as described previously [13]. Briefly, 4-μm sections were deparaffinised with xylene and rehydrated in graded concentrations of ethanol. Endogenous peroxidase was blocked in 0.3% H_2_O_2_ solution, and microwave antigen retrieval was performed in Citrate pH 6.0 (for HLA class I) or in Tris–EDTA pH 9.0 (for PD-L1). Subsequently, sections were incubated overnight at 4 °C with the different primary antibodies, as detailed in Table 2. The next day, the staining was detected using poly-HRP and visualised with a DAB+ substrate chromogen system. Slides were counterstained with haematoxylin, dehydrated and mounted with CV mount (Leica Microsystems). Tonsil normal tissue sections were used as positive controls, and primary antibodies were omitted for the negative controls. When loss of tissue occurred during the staining procedure, specimens were not included in the analysis.

**Table 2:** Characteristics of the antibodies and reagents used for immunohistochemistry (IHC) and immunofluorescence (IF)

Antigen	Antibody supplier	Clone/reference	Isotype	Antibody dilution	Secondary reagent	Reagent supplier	Reagent reference
IHC							
HCA2	Nordic-Mubio	MUB2036P	Mouse IgG1	1/3200	BrightVision	Immunologic	DPVO-HRP
HC10	Nordic-Mubio	MUB2037P	Mouse IgG2a	1/3200	BrightVision	Immunologic	DPVO-HRP
β2 m	Dako	A0072	Rabbit IgG	1/1600	BrightVision	Immunologic	DPVO-HRP
PD-L1	Cell signaling	E1L3 N	Rabbit IgG	1/400	BrightVision	Immunologic	DPVO-HRP
IF							
CD3	Dako	A0452	Rabbit IgG	1/400	GαR IgG-A546	Invitrogen	A11010
CD8	Novocastra	4B11	Mouse IgG2b	1/200	GαM IgG2b-A647	Invitrogen	A21242
FoxP3	Abcam	236A/E7	Mouse IgG1	1/100	GαM IgG1-A488	Invitrogen	A21121

*α* anti, *A* Alexa Fluor labelled, *G* goat, *M* mouse

**Fig. 1:**
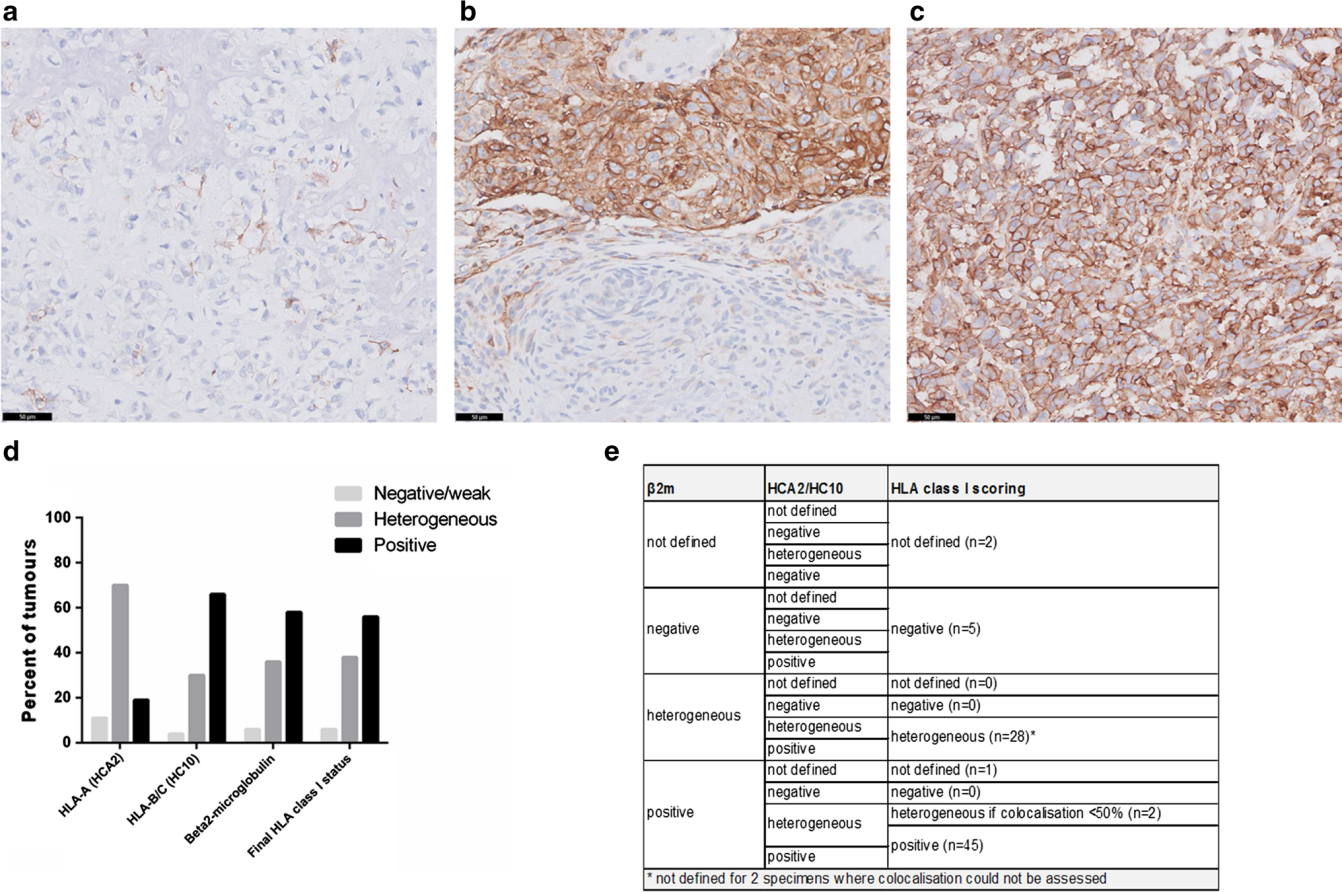
Different HLA class I phenotypes in osteosarcoma. Representative staining patterns of HLA-A expression using immunohistochemistry (HCA2 antibody): negative/weak expression with endothelial cells as positive internal controls (**a**), heterogeneous expression with both negative and positive regions (**b**) and diffuse positive expression (**c**). *Scale bars* 50 μm. Negative and heterogeneous expression was observed more frequently for HLA-A compared to HLA-B/C (**d**). The final HLA class I expression status was determined according to the positivity of at least one of the heavy chains stainings (either HCA2, HC10 or both), dependent to the β2-microglobulin positivity score (**e**)

Whole sections were scored independently by two observers (Y.T. Sundara and J.V.M.G. Bovée or A.H.G. Cleven). As semiquantitative scores were used, there was a high concordance between observers and in case of discrepancies, the slide was reviewed to reach a consensus. The expression of HLA-A (HCA2 staining), HLA-B/C (HC10 staining) and β2-microglobulin was assessed semiquantitatively as negative/focal weak (tumour cells negative or focally and weakly positive with positive internal controls), heterogeneous (both negative and positive regions on the same slide) and positive (moderate or strong staining in the whole tumour), as used in a previous study [13]. Because the light chain β2-microglobulin is an essential constant component of HLA class I molecules, the final HLA class I expression status was determined according to the positivity of at least one of the heavy chains stainings (either HCA2, HC10 or both), combined with the β2-microglobulin positivity score. Moreover, as many tumours were found heterogeneous for the different HLA stainings, we also assessed the degree of colocalisation between β2-microglobulin and HCA2/HC10. This HLA scoring is detailed in Fig. 1e. Positive PD-L1 expression was defined as ≥1% of tumour cells or immune cells showing a membranous staining of any intensity, according to published data [14]. Additionally, sections with heterogeneous expression of HLA class I were scanned using Panoramic MIDI scanner (3DHISTECH Ltd.) to assess colocalisation with T-cell infiltration.

### Immunofluorescent staining and scoring

For the detection and the characterisation of tumour-infiltrating T cells, we used a triple immunofluorescent staining, as previously described [15]. After antigen retrieval using Tris–EDTA buffer pH 9.0, whole sections were incubated overnight at 4 °C with the combination of primary antibodies, as detailed in Table 2. The following day, isotype-specific secondary antibodies labelled with Alexa fluorochromes (Life Technologies) were added during 1 h at room temperature and slides were mounted using Vectashield mounting medium containing DAPI (Vector Laboratories).

Stained sections were observed at 250× magnification using a confocal scanning microscope (Zeiss LSM 700), and four randomly selected images were processed with the ZEN software (version 2.1, Carl Zeiss). The numbers of CD3^+^CD8^−^ (membranous red staining), CD3^+^CD8^+^ (membranous purple staining) and CD3^+^CD8^−^FOXP3^+^ (green nuclear staining) T cells were counted using the cell counter plug-in of the program ImageJ version 1.48, and the results of the four images were averaged.

### Statistical analysis

All statistical analyses were performed using SPSS software version 23.0 (IBM Corporation, New York, USA), and graphs were constructed using GraphPad Prism software version 6 (La Jolla, California, USA). Correlations between immunohistochemical data and clinicopathological variables were analysed using Spearman’s rank correlation coefficient and Mann–Whitney *U* tests. For patients with material from different stages of the disease, paired *t* tests were used. A binary logistic regression model was used for predicting T-cell infiltration based on HLA class I expression. Correlations between immune markers and patient survival were tested using Kaplan–Meier survival analysis. Survival curves with immune data from primary tumours were generated based on biopsy specimens (16 patients), which reflect the natural host immune response and limit the impact of any neoadjuvant treatment on tumour microenvironment, or based on resection specimens when biopsy material was not available (six patients). Data from the first metastatic lesion were used for assessing overall survival from the time of diagnosis of metastatic disease (16 patients). *P* values below 0.05 were considered statistically significant.

## Results

### HLA-A is more frequently negative or heterogeneous than HLA-B/C in osteosarcoma

HLA class I expression was determined on whole tumour sections by immunohistochemistry using the antibodies HCA2 (HLA-A), HC10 (HLA-B/C) and β2-microglobulin. Expression of HCA2 and HC10 was mostly membranous while expression of β2-microglobulin was both membranous and cytoplasmic. Representative images of the different patterns of HLA class I expression (negative/weak, heterogeneous and positive) are shown in Fig. 1a–c and Supplementary Figure 1. A selective lower HLA-A expression was more frequently observed than HLA-B/C, with 11% (*n* = 9) of the tumours that did not or weakly expressed HLA-A and 70% (*n* = 57) displayed a heterogeneous expression versus 4% (*n* = 3) and 30% (*n* = 24), respectively, for HLA-B/C **(**Fig. 1d and Supplementary Table 1). Overall, HLA class I expression was strongly positive in 56% (*n* = 45), heterogeneous in 38% (*n* = 30), and negative or weakly positive in 6% (*n* = 5) of 80 evaluated tumours, using the scoring described in Fig. 1e. No significant difference in HLA class I expression was observed between primary tumours, relapses and metastases (*p* = 0.58), neither between pretreatment biopsies and surgical resections (*p* = 0.48). For the patients with metastases at diagnosis, the expression status of HLA class I in the primary tumour and associated synchronous metastasis was found similar while it mainly differed for the patients with metachronous metastases (Supplementary Table 2).

HLA class I expression was also evaluated on normal bone cells, when present. Osteocytes were mainly negative for both HLA-A and HLA-B/C, with a more variable expression of β2-microglobulin. A positive but weak expression of all markers was often detected on osteoblasts **(**Supplementary Figure 2).

### Density of tumour-infiltrating lymphocytes is higher in metastatic lesions

**Fig. 2:**
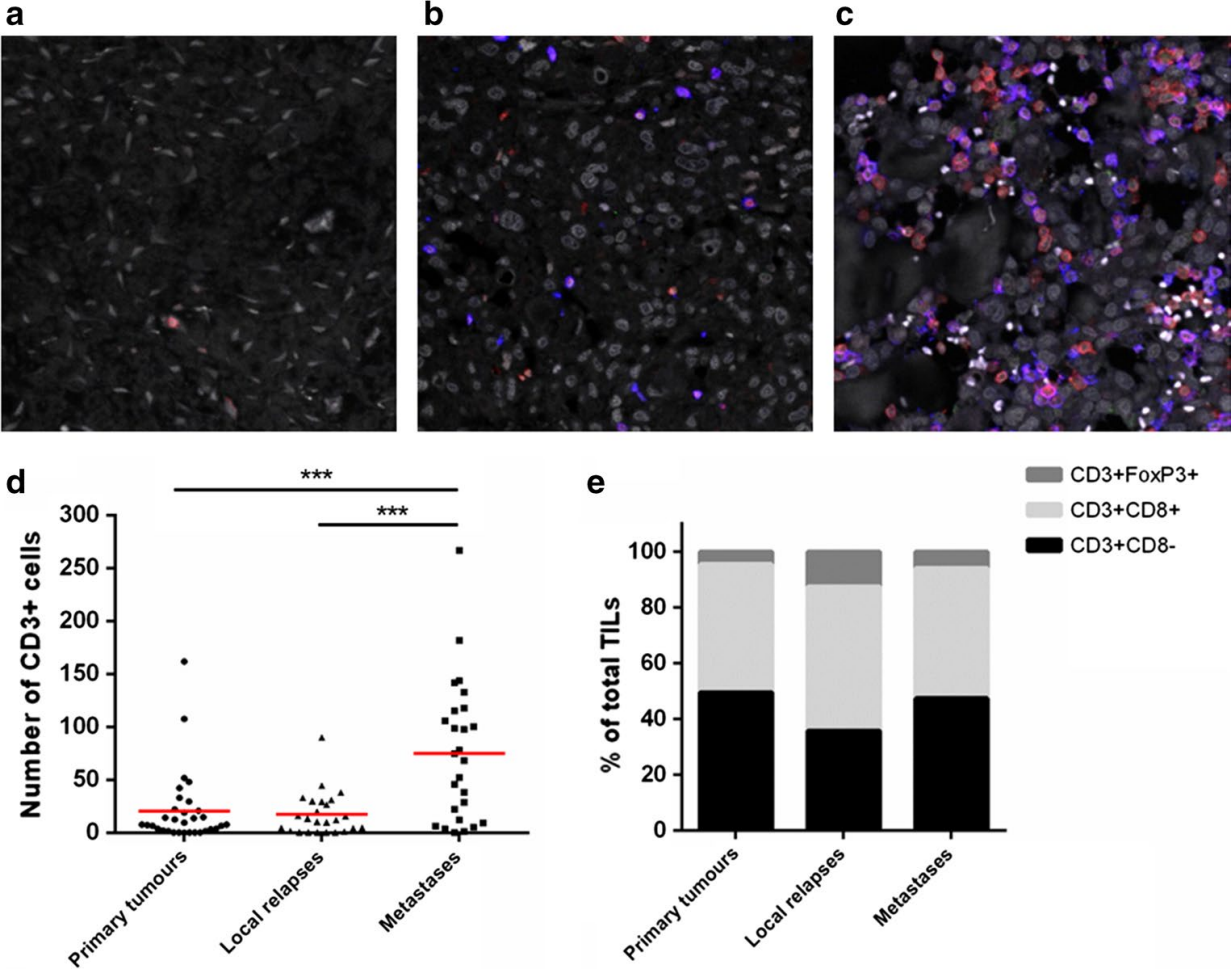
Characterisation of T-cell density and subtypes in osteosarcoma. Representative images for tumour-infiltrating lymphocytes in primary tumour (**a**) and the associated local relapse (**b**) and metastasis (**c**). Triple immunofluorescent staining using anti-CD3 (*red*), anti-CD8 (*blue*) and anti-FOXP3 (*green*) was used to identify CD3^+^CD8^−^, CD3^+^CD8^+^ and CD3^+^CD8^−^FOXP3^+^ T cells. Density of T-cell infiltration was higher in metastatic lesions (**d**), but the proportion of CD3^+^CD8^−^ and CD3^+^CD8^+^ T cells was comparable with primary tumours (**e**). CD3^+^CD8^−^FOXP3^+^ T cells were found more frequently in local relapses

The presence and type of tumour-infiltrating T cells were determined by a triple immunofluorescent staining (CD3-CD8-FOXP3) on whole osteosarcoma sections. Representative images are presented in Fig. 2a–c and Supplementary Figure 3. Density of T cells, defined as CD3 expressing cells, was significantly higher in metastatic lesions (mean ± SE = 75 ± 13 CD3^+^ cells) than in primary tumours (19 ± 6 CD3^+^ cells) and local relapses (18 ± 4 CD3^+^ cells) (*p* = 0.0003), as shown in Fig. 2d. In primary osteosarcomas, 46% of these tumour-infiltrating T cells were CD3^+^CD8^+^ T cells, 52% in local relapses and 47% in metastases. CD3^+^CD8^−^ cells (presumably composed of CD4^+^ and γδ T cells) constituted 49 and 47% of the T-cell infiltrate in primary tumours and metastases, while this proportion was observed lower (36%) but without statistical significance (*p* = 0.73) in local relapses (Fig. 2e and Supplementary Table 1). The local relapses displayed a higher proportion (12%) of CD3^+^CD8^−^FOXP3^+^ T cells, compared to primary tumours (4%; *p* = 0.15) and metastases (6%; *p* = 0.024). We did not observe CD3^+^CD8^+^FOXP3^+^ cells.

### Correlation between HLA class I expression and tumour-infiltrating T cells

We attempted to predict T-cell infiltration based on the expression of HLA-A, HLA-B/C and β2-microglobulin as a single independent variable or combined inter-dependent variables, using a binary logistic regression model. We found that the expression status of β2-microglobulin together with the expression of HLA-B/C was a significant predictor for the total T-cell count (*p* = 0.04), but not if the HLA-A expression status was added to the prediction model. Indeed, when we considered tumours with positive HLA class I expression including both HLA-A and HLA-B/C, the mean number of T cells (39 ± 7 CD3^+^ cells) was not significantly higher compared to tumours with heterogeneous (33 ± 10 CD3^+^ cells; *p* = 0.52) or negative HLA class I expression (25 ± 18 CD3^+^ cells; *p* = 0.43). However, T-cell infiltrate was highly heterogeneous within the tumours and we noticed in tumours with heterogeneous HLA expression that tumour areas expressing more β2-microglobulin and one of the HLA class I molecules contained more T cells than tumour areas with low expression of HLA class I. Consecutive cut sections stained for HLA class I and the triple CD3-CD8-FOXP3 combination were scanned using a Panoramic MIDI scanner in order to illustrate this observation of spatial colocalisation on selected areas, as shown in Supplementary Figure 4.

### High T-cell infiltrate associates with PD-L1 expression

**Fig. 3:**
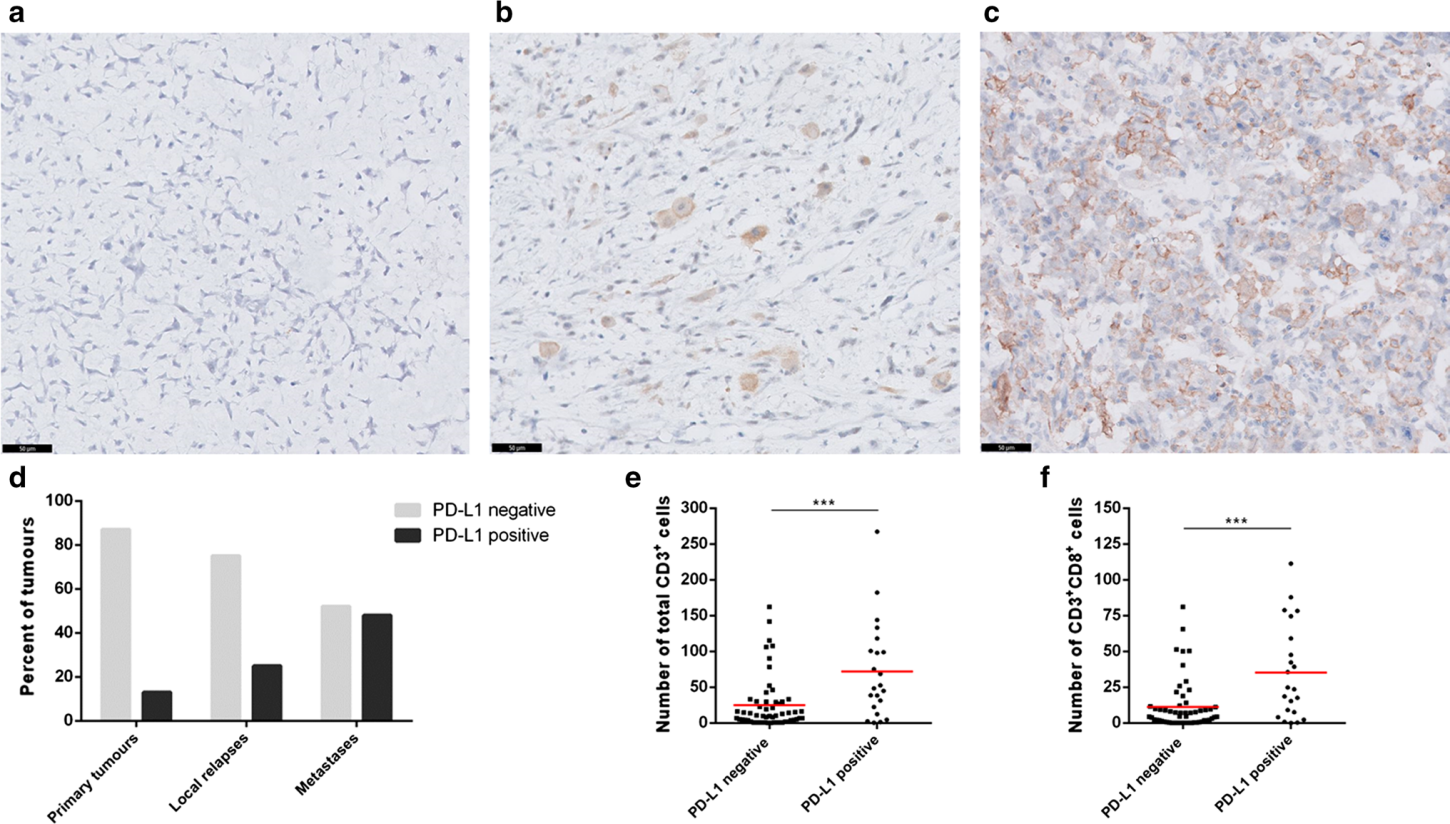
High PD-L1 expression in metastatic osteosarcoma lesions. Representative images for PD-L1 immunostaining in an osteosarcoma patient. PD-L1 was negative in the primary tumour (**a**) while a membranous expression was detected on isolated cells (mainly macrophages) in the local relapse (**b**) and was more diffuse and observed on both osteosarcoma cells and immune cells in the lung metastasis (**c**). *Scale bars* 50 μm. PD-L1 expression was observed more frequently in metastatic osteosarcoma lesions, compared to primary tumours and local relapses (**d**). T-cell infiltration was higher in PD-L1 positive tumours (**e**, **f**)

As PD-L1 expression is a possible mechanism for tumours to evade immune-mediated destruction, its expression was determined by immunohistochemistry on whole tumour sections. Membranous PD-L1 expression on ≥1% of osteosarcoma cells and/or immune cells (mainly macrophages) was found in 22 of 79 evaluated tumours (27.8%). Representative images for PD-L1 staining on a progressive disease are shown in Fig. 3a–c. PD-L1 positivity was significantly higher in metastatic lesions (48%) compared to local relapses and primary tumours (25 and 13%, respectively; *p* = 0.004) (Fig. 3d). High density of total CD3^+^ tumour-infiltrating T cells and CD3^+^CD8^+^ T cells correlated with PD-L1 expression (*p* = 0.002 and *p* = 0.001, respectively) (Fig. 3e, f). We also noticed a frequent spatial colocalisation between T cells and areas of the tumour with PD-L1 positivity. Six of the 17 patients with a known progressive disease stained positive for PD-L1 in metastases but not in primary tumours, and two patients had PD-L1 expression on both primary tumour and metastasis. PD-L1-positive metastastic lesions were significantly more infiltrated by T cells than PD-L1 negative lesions (mean ± SE = 123 ± 20 vs. 40 ± 19 CD3^+^ cells; *p* = 0.012).

### Prognostic significance of immune markers in primary tumours and metastases

**Fig. 4:**
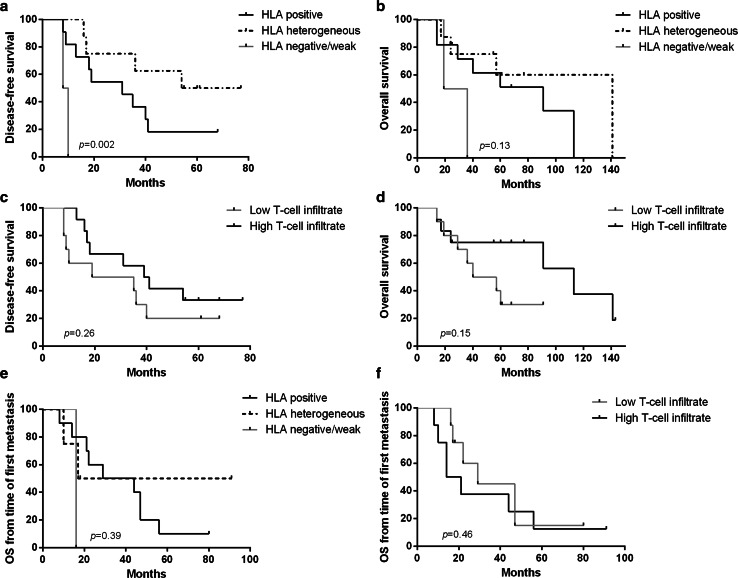
Correlation between HLA class I status, total T cells and patient survival. Kaplan–Meier survival curves for disease-free survival and overall survival according to HLA class I status (**a**, **b**) and density of T-cell infiltration (**c**, **d**) of the primary tumour. Overall survival from the time of diagnosis of metastatic disease according to HLA class I status (**e**) and T-cell infiltration (**f**) of the first metastatic lesion. *p* value obtained by log-rank test

Although mostly patients with poor outcome were included in this study, the time to progression differed between these patients, and there were also patients with non-progressive disease. Therefore, the prognostic significance of HLA class I expression, PD-L1 and T-cell infiltrate was assessed in the evaluable primary tumours (*n* = 22), as well as in the first metastatic lesion (*n* = 16), using Kaplan–Meier survival analysis. Heterogeneous and positive HLA class I expression in the primary tumour associated with a better disease-free survival compared to a negative/weak expression (*p* = 0.001 and *p* = 0.025, respectively; overall comparison between the three groups *p* = 0.002). However, a trend but no significant correlation was found for the overall survival (*p* = 0.13) (Fig. 4a, b). To evaluate the significance of infiltrating T cells, patients were divided in two groups based on the median numbers of total CD3^+^ cells (high and low). Patients with high T-cell infiltrate in the primary tumour tended to have a better clinical outcome although this was not significant. For this group of patients, the median disease-free survival was 39 versus 19 months for patients with low T-cell infiltrate (*p* = 0.26) and the difference in the median overall survival was more pronounced (112 vs. 40 months; *p* = 0.15) (Fig. 4d, e). Neither HLA class I expression nor density of T-cell infiltrate in the first metastatic lesion was related to overall survival from the time of diagnosis of the metastatic disease (Fig. 4c, f). PD-L1 status in either primary tumours or metastases did not correlate with patient survival (Supplementary Figure 5).

## Discussion

In this study, we investigated HLA class I and PD-L1 expression, as well as T-cell infiltration in osteosarcoma, to assess the potential application of T-cell-based immunotherapies. To characterise the HLA class I expression, we used the monoclonal antibodies HCA2 and HC10, which recognise HLA-A and HLA-B/C heavy chains, respectively, and an antibody specific for the light chain β2-microglobulin. Overall, we observed defects in HLA class I expression in 44% of the osteosarcomas, mainly with a heterogeneous pattern instead of a complete lack of HLA class I. Previous studies have reported a loss or a down-regulation of HLA class I expression in approximately 50–62% of osteosarcomas, using a pan-HLA class I antibody (EMR8-5) and a cut-off of 50% positive tumour cells [16, 17]. In our series, negative or heterogeneous expression of HLA-A molecules was found more frequently compared to the other heavy chains HLA-B/C and β2-microglobulin. The clinical and therapeutic relevance of this finding, if any, has to be further investigated, since HLA-A and HLA-B both are antigen presenting molecules, and required for T-cell-based immunotherapies.

Another major clinical finding in our series is the high density of tumour-infiltrating T cells in metastatic osteosarcoma lesions compared to primary tumours and local relapses. CD3^+^CD8^−^ and CD3^+^CD8^+^ T cells were found in the same proportion within the tumour microenvironment while the density of CD3^+^CD8^−^FOXP3^+^ T cells was higher in local relapses, probably reflecting local immune escape mechanisms. Expression of the light chain β2-microglobulin together with the HLA-B/C was an important predictor of T-cell infiltration. In other cancers, particularly with high mutational burden such as melanoma, lung and colorectal cancers, a high T-cell infiltrate commonly associates with better clinical outcome and may predict response to immunotherapy [10, 18, 19]. Although not statistically significant, high T-cell infiltrate in primary osteosarcomas also tended to have survival benefit for the patients included in our study. It has been demonstrated that tumour-infiltrating lymphocytes could be easily isolated from adult osteosarcomas and exhibited in vitro a high cytotoxic activity, suggesting that therapies based on these tumour-infiltrating T cells could be an efficient strategy in osteosarcoma [20]. However, some tumours are poorly infiltrated by T cells and the mechanisms associated with reduced T-cell trafficking and infiltration are poorly understood. A potential mechanism might be the loss of the tumour suppressor gene *PTEN*, frequently reported in osteosarcomas [21, 22].

PD-L1 positivity in metastases, while mainly negative in the associated primary tumours, emphasises the dynamics of an adaptive mechanism of immune escape. Overall, PD-L1 expression was found in almost half of the metastatic osteosarcoma lesions (48%), on both osteosarcoma cells and immune cells, mainly macrophages. Our results are in accordance with other groups who reported PD-L1 positivity using another monoclonal antibody only in metastatic osteosarcomas, as well as a higher PD-L1 mRNA expression in metastases, which correlated with the T-cell infiltrate [23, 24]. An increased PD-1 expression on peripheral CD4^+^ T cells was also observed in patients with metastases, strengthening the idea that PD-L1/PD-1 axis may play a role during osteosarcoma progression [25]. Recently, Koirala et al. reported the discrepancy between whole tumour sections and tissue micro-arrays as a result of the heterogeneity of PD-L1 expression in osteosarcoma, confirming our idea that this immune marker should be evaluated on whole sections [26]. Moreover, they identified PD-L1 positivity as a potential prognostic marker for poorer survival, which was not the case in our cohort. However, it should be noted that we mostly included patients with poor outcome, in order to assess the immune changes during disease progression. Therefore, our conclusions regarding the prognostic value of these different immune markers in primary tumours cannot be generalised in all osteosarcoma patients.

What is the explanation for the observed low and/or heterogeneous expression of HLA class I? In other cancers, defects in the antigen presentation pathway were reported at different levels (mutations in *HLA*-*A*, *HLA*-*B* and *HLA*-*C* genes, β2-microglobulin or defects in components of antigen-processing machinery) and have been regarded as mechanisms to escape from T-cell immune recognition [27–29]. Tumour cells that have a loss/down-regulation of HLA class I may gain a selective clonal advantage in a process called immunoediting, enabling them to escape from the CD8+ T-cell-mediated destruction [30].

However, in osteosarcoma, the question can be raised regarding the mechanisms involved in the frequent heterogeneous expression of HLA class I. First of all, there is scarce data on baseline HLA class I expression in normal bone cells. Therefore, it is not clear whether low HLA class I expression reflects the normal situation or, in contrast, ‘down regulation’ as a consequence of escape from T-cell immune recognition. An alternative explanation for heterogeneity of HLA class I expression could be the consequence of the extreme genomic instability of osteosarcoma. But another interesting interpretation would be the induction of HLA class I expression by interferon-γ secreted by T cells. In this study, we observed that in tumours with HLA class I negative and positive regions, the T cells spatially colocalised with the HLA class I positive tumour areas. Additionally, we observed a strong correlation between high numbers of tumour-infiltrating T cells and PD-L1 expression. Together, this could imply that, in areas of T-cell infiltration, immune activation leads to induction of HLA class I and PD-L1 expression. Although it would have been interesting to look at the difference in immune profile of primary tumour between patients with and without metastasis at diagnosis, we could not answer the question due to the low number of samples.

Importantly, and despite the limited number of patients, HLA class I expression in primary osteosarcomas correlated with a better disease-free survival, which is consistent with previous results [16, 17]. In addition, we observed that HLA class I expression in metastases was frequently positive, and this was in contrast to Tsukahara et al. [17] who reported that HLA class I loss or down-regulation occurred more commonly in metastatic osteosarcoma lesions (7 out of 8 cases; 88%). Consequently, the HLA data from our series can have two therapeutic implications. First, the frequent HLA class I expression in both primary tumours and metastases highlights the potential of (neo)antigen presentation by osteosarcoma cells, which can be exploitable in developing personalised immunotherapies. Second, identifying the rare patients with a negative or weak expression of HLA class I molecules on primary tumour is of clinical interest, to consider a different therapeutic approach (i.e. strategy based on NK cells) [31].

Altogether, the increased number of tumour-infiltrating T cells and PD-L1 expression during disease progression, associated with a frequent classical HLA class I expression, suggest that T-cell-based immunotherapy with adoptive cell transfer, peptide vaccines or immune checkpoint blockade could be a suitable treatment for metastatic osteosarcoma patients. Preclinical data demonstrated the benefit of PD-L1/PD-1 blockade antibodies, alone or in combination with anti-CTLA-4 in a mouse model of metastatic osteosarcoma and the efficacy of pembrolizumab, a monoclonal anti-PD-1 antibody, is currently being investigated for bone sarcomas in the phase II SARC028 study (NCT02301039) [23, 32]. Considering the limited therapeutic options currently available in advanced diseases, enhancing this pre-existing antitumour immune response in metastastic lesions may offer clinical benefit in osteosarcoma patients.

### Electronic supplementary material

Below is the link to the electronic supplementary material.

Supplementary material 1 (PDF 1445 kb)

## Abbreviations

CD: Cluster of differentiation
CTLA: Cytolytic T lymphocyte-associated antigen
DAB: 3,3′-diaminobenzidine
DAPI: 4′,6′-diamidino-2-phenylindole
EDTA: Ethylenediaminetetraacetic acid
FOXP3: Forkhead box p3
HLA: Human histocompatibility leucocyte antigen
HRP: Horseradish peroxidase
mRNA: Messenger RNA
NK cell: Natural killer cell
PD-1: Programmed death 1 receptor
PD-L1: Programmed death ligand 1
