# Supplementary material for: Increased PD-L1 and T-cell infiltration in the presence of HLA class I expression in metastatic high-grade osteosarcoma: a rationale for T-cell-based immunotherapy
Source: Cancer Immunol Immunother. 2016 Nov 16;66(1):119–28. doi: 10.1007/s00262-016-1925-3 (PMC5222929; doi:10.1007/s00262-016-1925-3)
Supplement: Supplementary file 1 — Supplementary material 1 (PDF 1445 kb) [file 262_2016_1925_MOESM1_ESM.pdf]

Supplementary Table 1. Overview of patient materials and staining results

Patient	Tissue-type	Disease-type	Time [a]	β2-microglobulin	HCA2	HC10	Final HLA score	CD3+CD8-	CD3+CD8+	CD3+FoxP3+	PD-L1
1	Biopsy	Primary Tumor	-	Positive	Positive	Not Defined	Positive	0	0	0	Negative
	Resection	Primary Tumor	3	Positive	Heterogeneous	Not Defined	Heterogeneous	8,75	4,25	1,5	Negative
	Biopsy	Metastasis	50	Positive	Heterogeneous	Positive	Positive	88,5	7,5	22	Positive
2	Biopsy	Primary Tumor	-	Heterogeneous	Heterogeneous	Positive	Heterogeneous	1	0,75	0	Negative
	Resection	Primary Tumor	5	Heterogeneous	Heterogeneous	Heterogeneous	Heterogeneous	2,75	9,75	0,25	Negative
	Biopsy	Local Relapse	36	Heterogeneous	Focal Weak	Positive	Heterogeneous	3,25	1,75	0	Negative
	Resection	Local Relapse	37	Heterogeneous	Focal Weak	Heterogeneous	Heterogeneous	0,5	0	0	Negative
	Resection	Metastasis	43	Positive	Heterogeneous	Heterogeneous	Positive	51	50,25	4,75	Negative
3	Resection	Primary Tumor	3	Positive	Heterogeneous	Positive	Positive	0,25	0,25	0	Not defined
	Resection	Primary Tumor	3	Positive	Heterogeneous	Positive	Positive	6,75	9	6,5	Positive
	Biopsy	Local Relapse	40	Positive	Heterogeneous	Positive	Positive	15,25	24,75	4,75	Positive
	Resection	Local Relapse	41	Positive	Heterogeneous	Positive	Positive	20,25	7	2,75	Negative
	Resection	Metastasis	53	Positive	Positive	Positive	Positive	10,5	35,5	6,5	Positive
	Resection	Metastasis	58	Positive	Heterogeneous	Positive	Positive	52,5	42,25	4	Positive
4	Biopsy	Primary Tumor	-	Focal Weak	Heterogeneous	Focal Weak	Negative	0,25	0,25	0	Negative
	Resection	Metastasis	9	Focal Weak	Not Defined	Heterogeneous	Negative	9	11,5	2	Negative
	Resection	Metastasis	16	Heterogeneous	Heterogeneous	Not Defined	Heterogeneous	2	4,5	0	Negative
	Biopsy	Metastasis	21	Positive	Heterogeneous	Positive	Positive	1,5	0	0	Negative
	Biopsy	Local Relapse	30	Positive	Positive	Positive	Positive	8,75	7	0,5	Negative
5	Resection	Primary Tumor	-	Positive	Heterogeneous	Heterogeneous	Positive	1	3	0	Negative
	Biopsy	Local Relapse	17	Positive	Heterogeneous	Focal Weak	Heterogeneous	0	0,25	1,5	Negative
	Resection	Local Relapse	19	Positive	Heterogeneous	Heterogeneous	Positive	0,5	0	0,75	Positive
	Resection	Local Relapse	30	Heterogeneous	Heterogeneous	Positive	Heterogeneous	8,75	9,25	8,75	Negative
	Resection	Metastasis	37	Heterogeneous	Heterogeneous	Heterogeneous	Heterogeneous	0	0,5	0	Negative
6	Biopsy	Primary Tumor	-	Positive	Heterogeneous	Positive	Positive	24,25	23,5	0,5	Positive
	Resection	Local Relapse	32	Heterogeneous	Heterogeneous	Positive	Heterogeneous	3,75	25,75	0	Negative
	Resection	Local Relapse	42	Heterogeneous	Heterogeneous	Heterogeneous	Heterogeneous	3,5	9,5	0,5	Negative
	Resection	Local Relapse	43	Heterogeneous	Heterogeneous	Positive	Heterogeneous	2	8	0	Negative
	Resection	Local Relapse	45	Positive	Heterogeneous	Positive	Positive	2	8,5	0	Negative
	Resection	Metastasis	87	Positive	Heterogeneous	Positive	Positive	20	47,5	1	Positive
7	Biopsy	Primary Tumor	-	Heterogeneous	Heterogeneous	Positive	Heterogeneous	33,5	65,5	8,75	Negative
	Resection	Primary Tumor	2	Positive	Heterogeneous	Positive	Positive	77,25	81	3,75	Negative
	Biopsy	Local Relapse	5	Focal Weak	Focal Weak	Heterogeneous	Negative	22,5	50	17,75	Negative
	Resection	Metastasis	6	Positive	Heterogeneous	Positive	Positive	12,25	17,5	8,75	Positive
	Resection	Metastasis	8	Not Defined	Not Defined	Not Defined	Not defined	19,5	74,5	6,5	Positive
	Resection	Metastasis	8	Heterogeneous	Not Defined	Heterogeneous	Heterogeneous	171	87,75	8,25	Positive
	Resection	Metastasis	10	Heterogeneous	Heterogeneous	Positive	Heterogeneous	17,75	39,25	18	Positive
8	Biopsy	Primary Tumor	-	Heterogeneous	Heterogeneous	Positive	Heterogeneous	2	2	0	Negative
	Resection	Primary Tumor	3	Heterogeneous	Heterogeneous	Heterogeneous	Not defined	6,75	7,25	0	Negative
	Resection	Metastasis	5	Positive	Heterogeneous	Positive	Positive	15	111,25	6,75	Positive
	Resection	Metastasis	7	Focal Weak	Heterogeneous	Heterogeneous	Negative	6,5	5,25	0,5	Not defined
	Resection	Local Relapse	8	Heterogeneous	Heterogeneous	Positive	Heterogeneous	5,25	14,5	0,5	Not defined
	Resection	Local Relapse	8	Heterogeneous	Heterogeneous	Heterogeneous	Heterogeneous	2,75	1,75	0	Negative

9	Resection	Primary Tumor	3	Positive	Focal Weak	Not Defined	Not defined	1,75	1,75	0	Negative
	Biopsy	Local Relapse	4	Heterogeneous	Heterogeneous	Positive	Heterogeneous	0,5	0	0	Negative
	Resection	Local Relapse	4	Positive	Heterogeneous	Heterogeneous	Positive	7	4	1,25	Positive
	Biopsy	Metastasis	6	Heterogeneous	Heterogeneous	Heterogeneous	Heterogeneous	56,75	78,25	8,75	Positive
10	Biopsy	Primary Tumor	-	Positive	Heterogeneous	Positive	Positive	0,25	0,25	0	Negative
	Resection	Local Relapse	34	Positive	Focal Weak	Positive	Positive	7	8,75	0,25	Negative
	Resection	Local Relapse	45	Positive	Heterogeneous	Positive	Positive	8	18,5	11,75	Positive
	Resection	Metastasis	91	Positive	Heterogeneous	Positive	Positive	97,75	78,75	5,5	Positive
11*	Resection	Metastasis	3	Positive	Positive	Positive	Positive	111,5	29	1,25	Negative
	Resection	Metastasis	9	Positive	Positive	Positive	Positive	61,5	51,25	2,5	Negative
	Resection	Metastasis	14	Positive	Positive	Positive	Positive	6	23	0	Negative
	Biopsy	Metastasis	37	Positive	Heterogeneous	Positive	Positive	2,5	0,5	0,75	Negative
	Resection	Local Relapse	38	Positive	Heterogeneous	Positive	Positive	12,25	15,25	3,75	Positive
12*	Biopsy	Primary Tumor	-	Positive	Positive	Positive	Positive	9	4,5	1,5	Negative
	Resection	Primary Tumor	2	Positive	Positive	Positive	Positive	5,25	0,75	0,75	Negative
	Resection	Metastasis	11	Not Defined	Positive	Not Defined	Not defined	35,25	59	3,5	Positive
	Biopsy	Local Relapse	12	Positive	Heterogeneous	Positive	Positive	0,5	0	0	Negative
13*	Biopsy	Primary Tumor	-	Positive	Positive	Positive	Positive	0,5	0	0	Negative
	Resection	Primary Tumor	2	Positive	Positive	Positive	Positive	2,5	3,75	0,5	Negative
	Resection	Local Relapse	3	Positive	Heterogeneous	Positive	Positive	2,25	2,25	0	Positive
	Biopsy	Metastasis	5	Positive	Heterogeneous	Positive	Positive	4	1,25	0	Negative
	Resection	Local Relapse	19	Positive	Positive	Positive	Positive	0,5	0	0	Negative
14	Biopsy	Primary Tumor	-	Positive	Heterogeneous	Positive	Positive	1,5	0,75	0	Positive
	Resection	Local Relapse	12	Positive	Positive	Positive	Positive	20,5	12	0,75	Negative
15*	Resection	Metastasis	4	Positive	Heterogeneous	Positive	Positive	31,75	14	0,25	Negative
16	Biopsy	Primary Tumor	-	Positive	Heterogeneous	Positive	Positive	23,75	9	0,5	Negative
17	Biopsy	Primary Tumor	-	Heterogeneous	Heterogeneous	Positive	Heterogeneous	20	18,75	3,75	Negative
	Resection	Primary Tumor	3	Positive	Positive	Positive	Positive	11,75	7,5	0	Negative
18	Resection	Primary Tumor	3	Positive	Positive	Positive	Positive	0	0	0	Negative
19	Biopsy	Primary Tumor	-	Heterogeneous	Heterogeneous	Positive	Heterogeneous	5,75	1,5	0,25	Not defined
	Resection	Primary Tumor	3	Heterogeneous	Heterogeneous	Heterogeneous	Heterogeneous	30,5	21,5	0	Negative
20	Biopsy	Primary Tumor	-	Heterogeneous	Focal Weak	Heterogeneous	Heterogeneous	18,75	11	0	Negative
	Resection	Primary Tumor	3	Heterogeneous	Focal Weak	Heterogeneous	Heterogeneous	14	7	0	Negative
21	Biopsy	Primary Tumor	-	Positive	Focal Weak	Positive	Positive	1,75	0	0	Not defined
	Resection	Primary Tumor	2	Positive	Heterogeneous	Heterogeneous	Positive	5,5	2,25	0,25	Negative
	Resection	Local Relapse	10	Heterogeneous	Heterogeneous	Heterogeneous	Not defined	0,5	0	0	Negative
22	Resection	Primary Tumor	2	Focal Weak	Heterogeneous	Heterogeneous	Negative	0,25	0,25	0	Not defined
	Resection	Metastasis	14	Heterogeneous	Heterogeneous	Heterogeneous	Heterogeneous	34,75	40,25	3,25	Negative
23	Biopsy	Primary Tumor	-	Heterogeneous	Positive	Positive	Heterogeneous	0	0,25	0	Positive
24*	Resection	Primary Tumor	3	Heterogeneous	Focal Weak	Heterogeneous	Heterogeneous	3,5	4,25	0,25	Negative
	Resection	Metastasis	6	Heterogeneous	Heterogeneous	Focal Weak	Heterogeneous	7	2	0,5	Negative
25	Resection	Primary Tumor	3	Heterogeneous	Heterogeneous	Heterogeneous	Heterogeneous	5,25	4	0,5	Negative

ª indicates time from initial diagnosis (months); * indicates metastasis present at the time of initial diagnosis

Supplementary Table 2. HLA class I status during osteosarcoma progression

	HLA class I expression status		
**Patient**	**Primary tumour**	**Local relapse**	**Metastases**
1	heterogeneous[a]	NA	positive[b]
2	heterogeneous	heterogeneous	positive
3	positive	positive	positive
4	negative[b]	positive[b]	negative<br>heterogeneous<br>positive[b]
5	positive	positive<br>heterogeneous	heterogeneous
6	positive[b]	positive	positive
7	positive[a]	negative[b]	positive<br>heterogeneous
8	heterogeneous[b]	heterogeneous	negative<br>positive
9	NA	positive	heterogeneous[b]
10	positive[b]	positive	positive
11[c]	NA	positive	positive
12[c]	positive	positive[b]	NA
13[c]	positive	positive	positive
14	positive	positive	NA
21	positive	heterogeneous	NA
24[c]	heterogeneous	NA	heterogeneous

[a] discrepancy between biopsy and surgical resection specimen
[b] determined on biopsy samples
[c] patient with metastatic disease at diagnosis
NA: not applicable

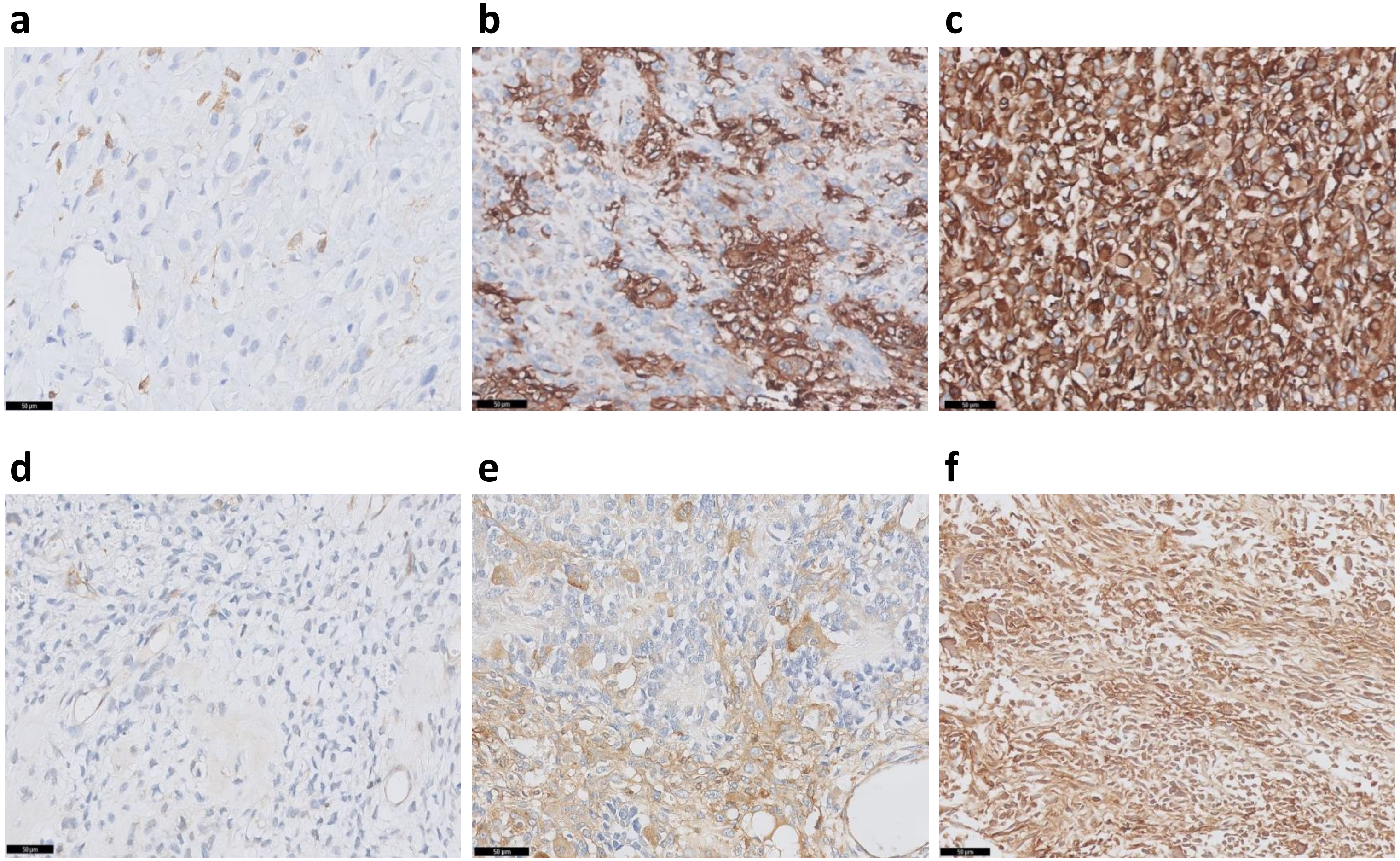

Supplementary Fig. 1 HLA-B/C and β2-microglobulin immunostaining in osteosarcoma

Representative staining patterns of HLA-B/C (**a-c)** and β2-microglobulin (**d-f**) expression using immunohistochemistry: negative/weak expression with endothelial cells as positive internal controls (**a,d**), heterogeneous expression with both negative and positive regions (**b,e**) and diffuse positive expression (**c,f**). Scale bars, 50μm.

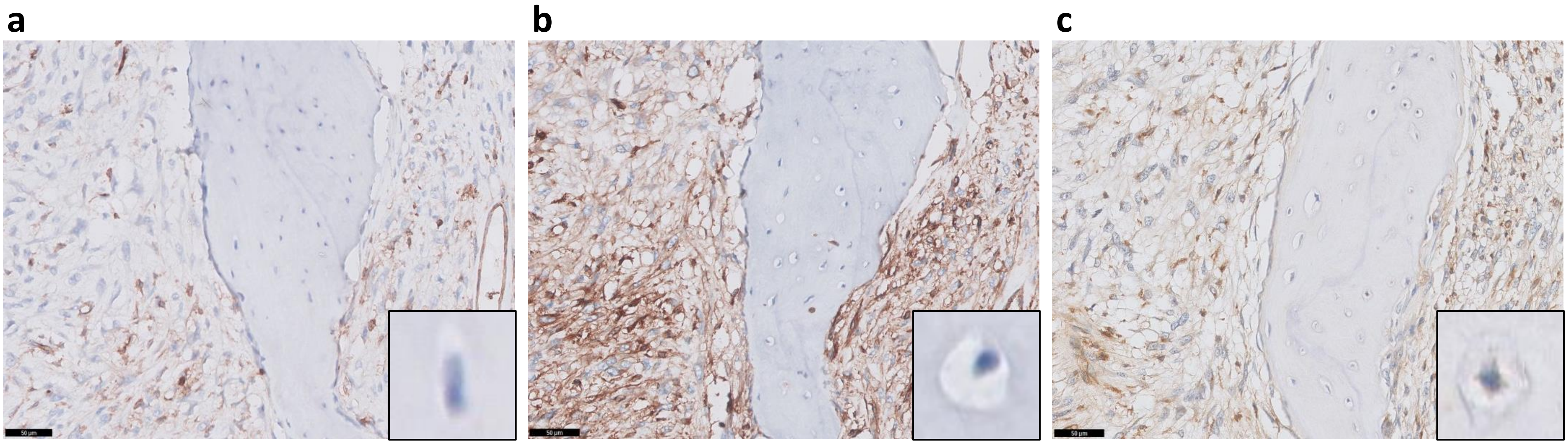

Supplementary Fig. 2 HLA class I expression in bone cells

Representative images of HLA-A (**a**), HLA-B/C (**b**) and β2-microglobulin (**c**) staining on normal bone, when present in tumour sections. Scale bars, 50µm.

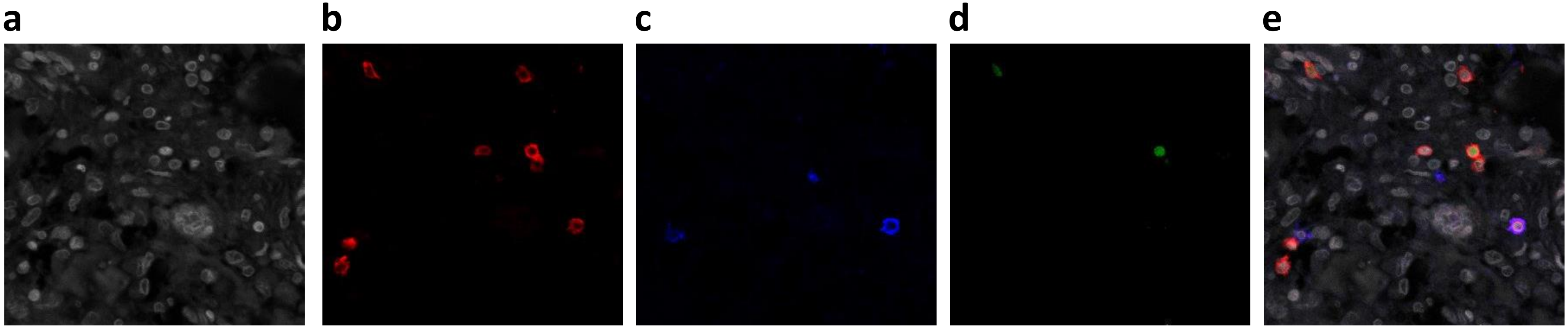

Supplementary Fig. 3 Immunofluorescent staining for T-cell characterisation

Representative images of primary osteosarcoma stained for DAPI (grey, **a**), CD3 (red, **b**), CD8 (blue, **c**), FOXP3 (green, **d**) and the merged picture (**e**). $CD3^{+}CD8^{-}$ T cells are characterised by a membranous red staining, $CD3^{+}CD8^{+}$ T cells by a membranous purple staining and $CD3^{+}CD8^{-}FOXP3^{+}$ T cells by a nuclear green staining associated with a membranous red staining. We did not observed cells double positive for CD8 and FOXP3.

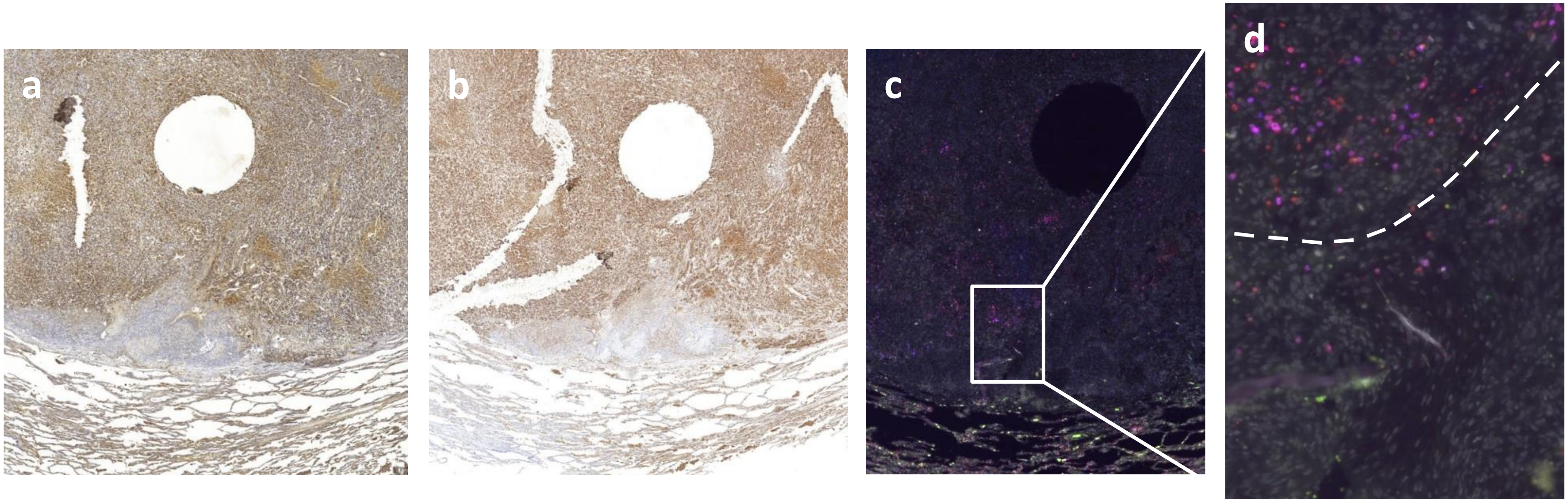

Supplementary Fig. 4 HLA class I expression colocalised with T-cell infiltration

Tumour areas with HLA class I positivity were frequently highly infiltrated with T cells compared HLA negative regions. Consecutive sections were stained for HLA class I using β2-microglobulin (**a**), HCA2 (**b**) and HC10 antibodies, then scanned using a Philips Ultra-Fast Scanner 1.6 RA. Triple fluorescent staining CD3-CD8-FOXP3 was used to detect and characterise T cells and the slides were scanned using a Panoramic MIDI scanner (**c,d**) to assess colocalisation with HLA class I positive regions.

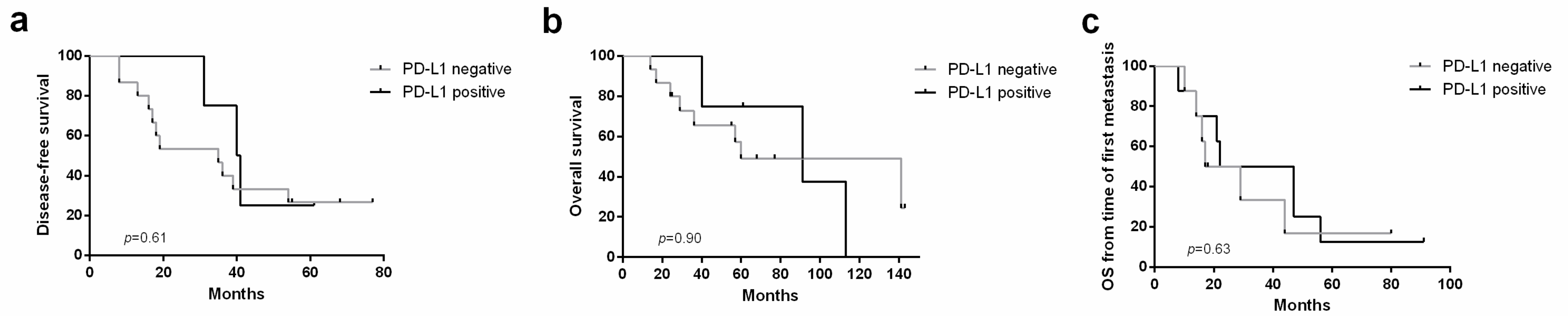

Supplementary Fig. 5 No correlation between PD-L1 expression and patient survival

Kaplan-Meier survival curves for disease-free survival (**a**) and overall survival (**b**) according to PD-L1 expression in the primary tumour, and overall survival from time of first metastasis (**c**) according to PD-L1 status in the first metastatic lesion. *P* value obtained by log-rank test.
